# Experimental Study on the Evolution of Mechanical Properties and Their Mechanisms in a HTPB Propellant Under Fatigue Loading

**DOI:** 10.3390/polym17202756

**Published:** 2025-10-15

**Authors:** Feiyang Feng, Xiong Chen, Jinsheng Xu, Yi Zeng, Wei Huang, Junchao Dong

**Affiliations:** 1 Key Laboratory of Special Engine Technology, School of Mechanical Engineering, Nanjing University of Science and Technology, Nanjing 210094, China; fengfeiyang@njust.edu.cn (F.F.); xujinsheng@njust.edu.cn (J.X.); zyworks79@yeah.net (Y.Z.); huangwei91@njust.edu.cn (W.H.); 2 Reliability Center, Beijing Institute of Strength and Environment, Beijing 100076, China; dongjunchaook@163.com

**Keywords:** HTPB propellant, fatigue loading, residual elongation, evolution model

## Abstract

In this study, we explored the evolution of mechanical properties in hydroxyl-terminated polybutadiene (HTPB) propellants under fatigue loading by performing fatigue tests with varying maximum stresses and cycle numbers, followed by uniaxial tensile tests on post-fatigue specimens. Residual elongation was used as a key parameter to characterize mechanical behavior, while scanning electron microscopy (SEM) provided insights into the mesostructural morphological changes that occur under different loading conditions, revealing the mechanisms responsible for variations in mechanical properties. The results show that, as the number of loading cycles increases, residual elongation decreases, with three distinct phases of decline—slow change, gradual decline, and rapid deterioration—depending on the stress levels. SEM analysis identified damage mechanisms such as “dewetting” and particle fragmentation at the mesostructural level, which compromise the material’s structural integrity, leading to reduced residual elongation. A novel aspect of this study is the application of Williams–Landel–Ferry (WLF) theory to construct a master curve describing residual elongation decay. This approach enabled the development of a generalized model to predict the material’s degradation under fatigue loading, with experimental validation of the fitted evolution model, offering a new and effective method for assessing the long-term performance of HTPB propellants.

## 1. Introduction

Solid rocket motors are widely used in the national defense and aerospace industries due to their simple structure, long-term storage capabilities, and rapid response characteristics [1,2]. Solid propellants, as composite materials based on polymers, are the primary energy source in these motors, and their stability ensures reliable performance in rocket propulsion systems [3,4]. However, solid rocket motors are subjected to various alternating loads during their operational lifespan, including vibrational and thermal cycling loads, which can significantly affect the integrity of the motor [5,6,7]. Due to the inherent viscoelastic nature of solid propellants, fatigue loading does not cause immediate macroscopic failure but instead leads to gradual internal damage and the continuous degradation of mechanical properties over time [8,9]. This cumulative damage ultimately results in abnormal operation or even failure of the rocket motor, posing significant safety risks. Therefore, investigating the evolution of mechanical properties in solid propellants under fatigue loading is crucial in guaranteeing the safety and reliability of rocket motors.

Research on solid propellant materials under fatigue loads has progressed significantly. Early studies focused on the stress–strain response under uniaxial fatigue loads, establishing models that correlated load frequency, stress ratio, and cycle count with fatigue life. These studies typically identified a pathway of “cyclic softening–damage accumulation–fracture failure”. For example, Gligorijević et al. [10] found a correlation between tensile properties and cumulative damage in propellants. As research advanced, scholars extended their focus to more realistic conditions, such as multiaxial and thermo-mechanical fatigue, and investigated macroscopic performance parameters such as elastic modulus degradation and Poisson’s ratio changes. These studies contributed to fatigue life prediction methods based on energy dissipation and damage mechanics.

More recently, research has shifted toward understanding the fatigue damage mechanisms and the relationship between microstructural changes and macroscopic performance. Secor [11] demonstrated that the fracture behavior under dynamic fatigue loads differs from that under static conditions. Meanwhile, advanced in situ techniques and multi-scale simulations have been used to explore how microdamages (e.g., interface debonding) influence macroscopic properties. Additionally, studies on novel high-energy solid propellants, including those with nano-additives, have examined their impact on fatigue resistance. Despite these advancements, challenges persist, particularly in extreme conditions characterized by wide temperature ranges and high strain rates, as well as under the combined effects of aging and fatigue damage. Furthermore, research using residual elongation as a fatigue indicator (e.g., Drozdov [12]; Starkova et al. [13]) provides valuable insights for assessing the fatigue behavior of solid propellants, including HTPB-based composites.

A significant body of research has explored the mechanical behavior of solid propellants under fatigue loading, utilizing both experimental and theoretical approaches. Xu et al. [14] examined the fatigue properties of HTPB-based propellants, studying their thermodynamic responses under different stress and frequency conditions. They analyzed the effects of damage on mechanical properties using uniaxial tensile tests and SEM observations after cyclic loading. Tong et al. [15,16] quantitatively assessed the effects of strain levels and frequency on the mechanical property degradation of HTPB propellants, constructing a viscoelastic–hyperelastic model to describe their response under cyclic loading. Additionally, studies by Hu et al. [17] and Li et al. [18] investigated fatigue behavior in NEPE and composite solid propellants, leading to the development of models to predict fatigue life and investigate the effects of strain-controlled fatigue on material properties. Other studies have also highlighted the influence of temperature changes induced by cyclic loading on the mechanical behavior of solid propellants, as well as the relationship between creep and fatigue [19,20,21,22].

In the context of multi-scale mechanics, Zhang et al. [23] and Picquart et al. [24] explored the non-linear viscoelastic behavior of solid propellants under cyclic loading, uncovering valuable insights into the microscopic mechanisms of fatigue damage and its influence on macroscopic properties. Liu et al. [25] applied synchrotron radiation X-ray imaging technology to observe internal damage in solid propellants, integrating these observations into a high-fidelity finite element model to analyze deformation and damage evolution on multiple scales. Zhang et al. [26] examined the effects of low-frequency strain loading on the residual strain accumulation and stress softening behavior of NEPE solid propellants, which enables a detailed understanding of fatigue-induced damage mechanisms.

Extensive research has been conducted on the various mechanical properties of solid propellants under fatigue loading. However, one key parameter—residual elongation—remains underexplored. Residual elongation serves as a critical indicator of plastic deformation capacity after fatigue damage, offering valuable insights into the structural integrity and long-term behavior of materials. While much of the existing research on residual elongation has focused on metallic materials, such as studies by Hua et al. [27] and Zhang et al. [28] on stainless steel and high-strength steel, few studies have investigated this parameter in the context of solid propellants. This is particularly relevant in the assessment of solid rocket motor grains, where residual elongation directly correlates with the material’s ability to withstand continued operational stresses.

In contrast with other mechanical parameters, such as dynamic modulus or stress softening, which have been widely used in previous studies, residual elongation provides a more direct measure of how a propellant material responds to long-term fatigue damage, serving as a reliable metric for evaluating the service life and overall structural integrity of solid propellants. Unlike dynamic modulus, which is more sensitive to short-term loading conditions, residual elongation captures the cumulative, long-term effects of fatigue, which is critical in ensuring the durability and safety of solid rocket motors. Therefore, in this study, we focused on residual elongation as the optimal parameter for characterizing fatigue-induced degradation in solid propellants.

This study aimed to fill this gap in the literature by conducting a series of fatigue tests on HTPB propellant specimens under varying stress levels and loading cycles. Following the fatigue tests, standard tensile tests were performed to determine the residual elongation of the specimens. The evolution of residual elongation was analyzed, and the variation mechanism was explored at the mesoscopic scale. A corresponding evolution model was developed to investigate further how residual elongation can serve as a predictive tool for assessing the mechanical behavior and service life of solid rocket motor grains. The findings of this study will provide essential insights into the structural integrity of solid propellants, which will have significant implications for the design, operation, and safety of solid rocket motors.

## 2. Experimental Setup and Method

### 2.1. Setup and Specimens

**Figure 1:**
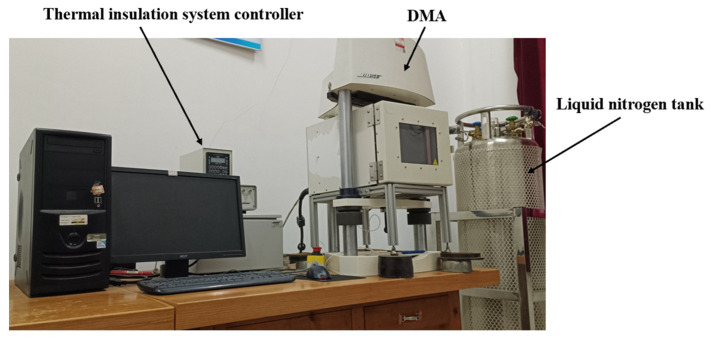
Dynamic mechanical analyzer and thermal control system.

In this study, both the fatigue tests and the standard tensile tests were conducted using the ELF3200 dynamic mechanical analyzer (DMA), manufactured by BOSE Corporation (Framingham, MA, USA). This instrument provides high-precision data storage and excellent load control performance, with an operating frequency range from 10^−4^ Hz to 200 Hz. Additionally, the experimental setup includes a thermal insulation system from SUN Corporation (Titusville, FL, USA) and a liquid nitrogen tank, which provides a stable temperature environment for the experiment. The experimental equipment is shown in Figure 1.

The specimens comprised hydroxyl-terminated polybutadiene (HTPB) propellants. Detailed information regarding the main components of HTPB propellants is provided in Table 1. Given the sensitivity of HTPB propellants to environmental conditions, especially humidity, the raw billet material was continuously sealed and stored in a dry box for 24 h before testing to ensure the stability of its properties. All production and testing processes were carried out in a controlled environment at constant temperature and humidity to minimize any fluctuations that might affect material performance.

**Figure 2:**
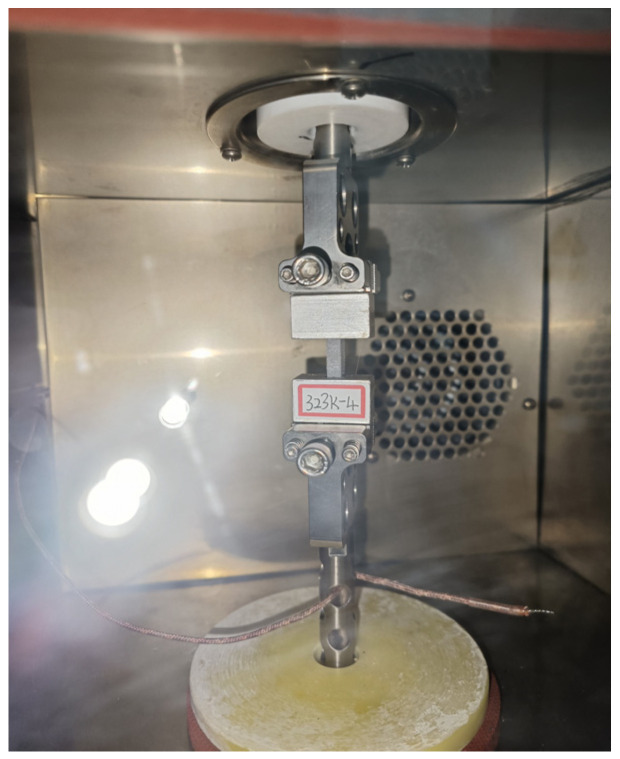
Connection method between the fixture and DMA.

The specimens were 33 mm in length, 10 mm in width, and 5 mm in thickness. To enhance the reliability of the test results, dedicated test fixtures were fabricated from aluminum alloy. The specimens were securely bonded to these fixtures using high-strength AB adhesives, which are particularly effective for bonding polymers to metals. The adhesive was cured for 24 h to ensure complete bonding before the specimen-fixture assembly was connected to the DMA controller’s fixture, as illustrated in Figure 2.

To ensure material consistency and environmental stability among the HTPB (hydroxyl-terminated polybutadiene) propellants, a rigorous quality control protocol was implemented. Given the complex composition of these propellants, which includes HTPB prepolymer and various curing agents, strict sourcing procedures were established. Raw materials were procured exclusively from certified and reliable suppliers with a proven track record. Upon receipt, each batch underwent essential quality checks to ensure consistency across batches. A dual-dimensional validation system was employed to comprehensively assess material variability, which incorporated both inter-batch and intra-batch verification. Inter-batch validation involved random sampling from multiple production lots to confirm consistency in material properties and prevent large-scale variations that could affect propellant performance. Each sample was tested against predefined specifications to identify any deviations in critical characteristics, although no testing of key parameters such as hydroxyl value, viscosity, or molecular weight was performed.

The intra-batch variability was evaluated through longitudinal testing at different time intervals. Throughout the lifecycle of each batch, samples were periodically extracted and analyzed for changes in key parameters, with residual elongation as the primary indicator. This approach enabled the detection of gradual material shifts or environmental influences—such as humidity and temperature—that could alter material properties. This dual-dimensional validation system facilitated the early detection of inconsistencies or deviations from expected performance, thereby ensuring the reliability and reproducibility of HTPB propellants throughout the study. By integrating random sampling with continuous temporal monitoring, the quality control process effectively minimized material variability, thus guaranteeing consistent specimen quality for fatigue testing and performance evaluation.

### 2.2. Experimental Method

**Figure 3:**
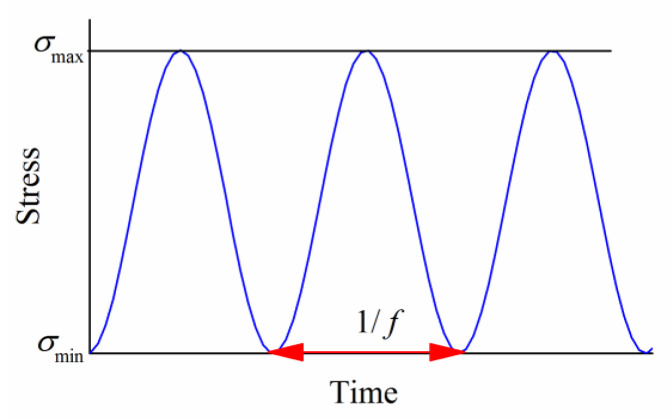
Stress-loading waveform for fatigue testing.

The fatigue testing of the HTPB propellant was conducted using a stress-controlled uniaxial sinusoidal loading method. The stress loading waveform is illustrated in Figure 3. The stress ratio R, defined as $R=\sigma_{min}/\sigma_{max}$, was set to 0.

The real-time stress during the fatigue process is given by Equation (1):

(1) $$\sigma =\frac{\sigma_{max}+\sigma_{min}}{2}.\left[1+sin\left(2\pi ft-\frac{\pi }{2}\right)\right]$$

where *σ* represents the real-time stress; $\sigma_{max}$ is the maximum loading stress; $\sigma_{min}$ is the minimum loading stress, which equals 0; the stress amplitude $\sigma_{a=}\sigma_{max}/2$; *f* is the loading frequency; and *t* represents time.

**Figure 4:**
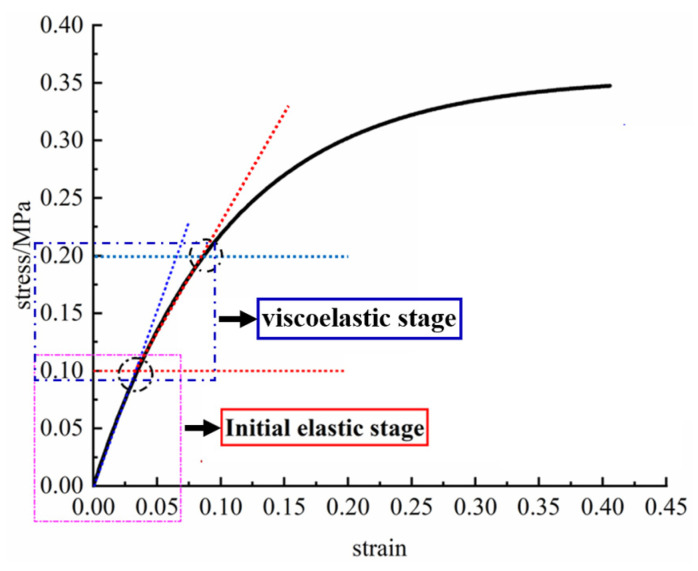
Standard tensile curve of HTPB propellant.

**Figure 5:**
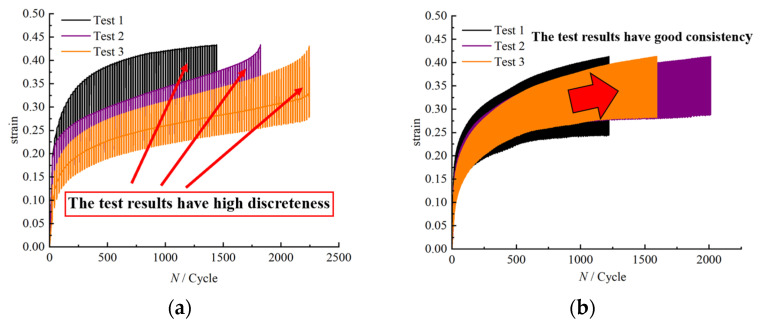
Variation in strain under different maximum loading stresses: (**a**) 0.18 MPa; (**b**) 0.17 MPa.

To determine the maximum loading stresses during fatigue testing, we referred to Figure 4, which presents the average stress–strain curves of HTPB propellant specimens obtained from repeated tensile tests conducted at 323 K with a tensile rate of 100 mm/min. As they comprise a typical viscoelastic material, HTPB propellants gradually accumulate damage under long-term cyclic loading. Selecting a stress range within the viscoelastic phase ensures that the fatigue tests realistically simulate service conditions. From the curve, the propellant enters a distinct viscoelastic phase at approximately 0.1 MPa, where non-linear effects become significant. Therefore, 0.1 MPa was selected as the lower limit of the maximum loading stress range. The propellant begins to yield at approximately 0.2 MPa, so, to ensure testing stability and result reliability, the maximum stress should not exceed this value. Preliminary tests showed that at 0.18–0.2 MPa, repeated fatigue tests exhibited high variability. For example, at 0.18 MPa, strain variation with loading cycles (Figure 5a) showed considerable scatter, whereas at 0.17 MPa, the test repeatability improved significantly (Figure 5b). Based on these observations, the maximum experimental stress level was determined to be 0.17 MPa, resulting in a fatigue testing stress range of 0.1–0.17 MPa.

The scatter observed in Figure 5, with greater dispersion at higher stress levels and smaller dispersion at lower stress levels, can be attributed to several factors: (1) material heterogeneity, including differences in microstructure such as grain size or defect distribution, which lead to variations in strain response under identical loading conditions; (2) the experimental equipment and operation, as loading precision, mechanical stability, and inconsistencies in specimen mounting can affect test results; and (3) environmental factors, including variations in temperature and humidity during testing, which can alter the mechanical behavior of the propellant, thereby increasing measurement variability.

Within the predefined stress range (0.1–0.17 MPa), representative values of 0.1, 0.14, and 0.17 MPa were selected to correspond to low, medium, and high stress levels, respectively. Subsequently, the fatigue life at each stress level was determined through testing, and the results are presented in Table 2. Based on these data, the fatigue testing protocols outlined in Table 3 were established, encompassing 18 experimental sets, each repeated three times under identical conditions to ensure reliability.

Throughout the fatigue tests, the temperature was maintained at 323 K, and the loading frequency was maintained at 5 Hz. These specific conditions were carefully selected to simulate realistic service environments for HTPB propellants, with a particular focus on replicating typical thermal and dynamic stresses encountered during high-temperature storage or early operational stages (e.g., heating before ignition).

The 323 K temperature is representative of in-service conditions where the propellant may be exposed to elevated temperatures, such as during storage in hot environments or while in transit before activation. This temperature ensures that the test results are reflective of the propellant’s behavior under realistic thermal loading scenarios.

The loading frequency was set to 5 Hz, primarily based on the mid-to-low frequency dynamic load characteristics that the propellant endures under operating conditions such as road transportation, maritime transportation, and engine operational vibration. Furthermore, preliminary tests revealed that a frequency of 5 Hz ensures test efficiency while preventing a significant temperature increase in the propellant during fatigue cycles, ensuring that the observed degradation is attributable to the propellant’s fatigue processes rather than test-induced heating.

By conducting fatigue tests at multiple stress levels and with different numbers of loading cycles, we were able to construct a comprehensive master curve of residual elongation decay, capturing the three-stage degradation process typically observed in solid propellants. This process included the slow-change, gradual-decline, and rapid-decline phases, providing a detailed, systematic understanding of how the material’s mechanical properties evolve under the typical service conditions. This master curve served as a reliable tool for predicting the long-term performance and failure risks of HTPB-based propellants, ensuring that the results apply to real-world usage scenarios.

The combination of 323 K temperature and 5 Hz frequency accounted for the temperature- and frequency-sensitive viscoelastic behavior of the material, ensuring that our tests accurately captured the degradation behavior in conditions relevant to actual service environments.

To eliminate the influence of residual thermal stresses on HTPB propellant specimens after fatigue testing, all samples were placed in a dry box with controlled temperature and humidity for 48 h. Subsequently, specimens subjected to different fatigue conditions were tested using standard tensile tests. The tests were performed at a constant rate of 100 mm/min using DMA equipment, with each specimen continuously stretched until rupture, marking the completion of the measurement.

### 2.3. Quality Control and Statistical Analysis

To enhance the reliability of our results, we implemented rigorous quality control measures throughout the study. These included ensuring consistency across sample batches, conducting pre-test conditioning, and calibrating instruments before testing. Replicate testing was performed to confirm the reproducibility of the results, and outlier exclusion criteria were applied to minimize potential experimental artifacts. Additionally, dimensional checks were conducted both before and after testing to verify specimen integrity.

For statistical analysis, we applied a series of procedures to enhance the robustness of our findings. Firstly, to assess the normality of data distributions, we used the Shapiro–Wilk test, which is preferred for small-to-moderate sample sizes. If the data did not meet the normality assumption (*p* ≤ 0.05), we applied a log transformation. If the transformed data still did not achieve normality, we used non-parametric tests such as the Kruskal–Wallis H test.

Outliers were identified using the interquartile range (IQR) method, with data points falling outside the range of Q3 + 1.5 × IQR or Q1 − 1.5 × IQR removed, provided that they also exceeded the mean ± 2.5 standard deviation. Following outlier removal, we reassessed normality to ensure that the assumptions for parametric tests were met.

To address variability across different stress levels, which could arise from repeated measurements and baseline differences, we employed a Linear Mixed-Effects Model (LMM). In this model, the stress level was treated as a fixed effect, whereas individual specimens were treated as a random effect to account for inter-specimen variability. This approach helped us to accurately estimate the impact of stress level while controlling for variability due to particular subjects.

All statistical tests were performed with a significance threshold of *p* < 0.05, and post hoc analyses were conducted when necessary. Power analysis was also carried out to ensure an adequate sample size, aiming for 80% power to detect meaningful effects. These comprehensive steps strengthened the transparency and reproducibility of our results.

## 3. Results and Discussion

### 3.1. Analysis of the Evolution Characteristics of Residual Elongation

Following fatigue testing, HTPB propellant specimens were evaluated using standard tensile tests to measure the elongation of the material at fracture. This measurement reflects the capacity for plastic deformation that remains after the material has sustained fatigue damage, quantitatively expressed as the residual elongation. A statistical analysis of repeated test results under various loading conditions was conducted to ascertain the average residual elongations across these conditions and the ratio of decline in these values compared to the maximum elongation of non-fatigued specimens. This decay ratio, referred to here as the residual elongation decay ratio, is detailed in Table 4.

The decay ratio ($\Delta \varepsilon_{b}$) is calculated using the following Equation (2):

(2) $$\Delta \varepsilon_{b}=\frac{\varepsilon_{0}-\varepsilon_{i}}{\varepsilon_{0}}$$

Here, $\varepsilon_{0}$ represents the maximum elongation measured in non-fatigued HTPB propellant specimens via tensile tests, with a mean value of approximately 40%, and $\varepsilon_{i}$ represents the residual elongation measured under different fatigue loading conditions.

**Figure 6:**
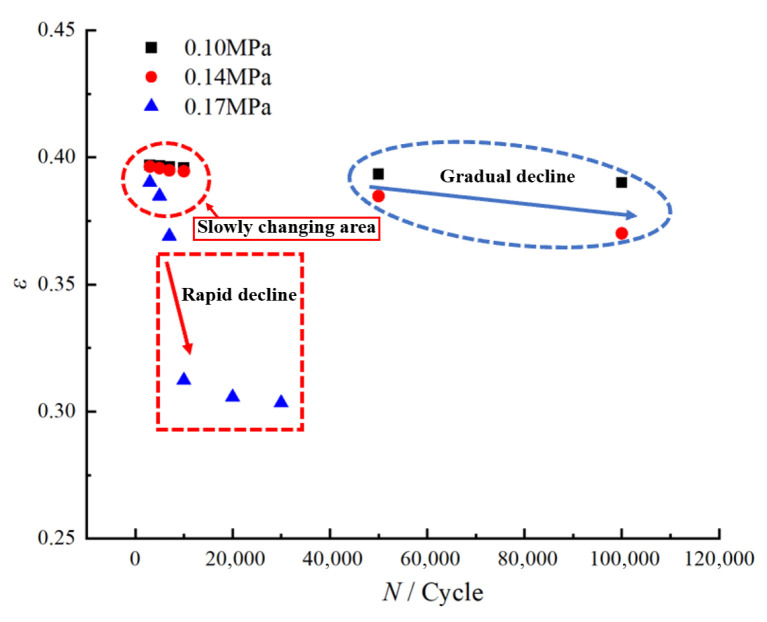
Distribution of residual elongation rates of HTPB propellant under various loading conditions.

Based on the aforementioned experimental data, Figure 6 illustrates the evolution of residual elongation in HTPB propellants under different loading conditions.

As shown in Figure 6, the evolution of residual elongation in HTPB propellant can be divided into three distinct regions, the “slow-change region”, the “gradual-decline region”, and the “rapid-decline region”, each corresponding to characteristic loading conditions. In the initial stage of fatigue loading, regardless of the maximum applied stress, the residual elongation remains relatively stable, defining the “slow-change region”. During this phase, the material maintains a high level of residual elongation, indicating that HTPB propellant preserves its mechanical integrity under a limited number of cyclic loadings. This behavior can be attributed to the inherent anti-fatigue properties of the HTPB propellant. At this stage, the viscoelastic network within the polymeric matrix remains largely intact, allowing the material to effectively absorb and buffer stress and strain through elastic and plastic deformation, resulting in only minor reductions in residual elongation as the number of cycles increases.

As fatigue loading continues, at low and medium stress levels of 0.10 MPa and 0.14 MPa, the residual elongation begins to enter the “gradual-decline region” after approximately 50,000–100,000 cycles. This region exhibits a more pronounced downward trend in residual elongation compared to the initial stage, reflecting the progressive degradation of mechanical properties under prolonged, low-stress fatigue conditions. Specifically, at 0.10 MPa, the residual elongation decreases gently, whereas at 0.14 MPa, the reduction becomes more significant, suggesting that higher loading stress accelerates the loss of plastic deformation capacity during fatigue.

When the maximum loading stress reaches 0.17 MPa, the residual elongation exhibits markedly different behavior. After only 7000 cycles, a rapid decrease is observed, corresponding to the “rapid-decline region” depicted in Figure 6. This rapid drop is the result of severe damage to the structural integrity of the HTPB propellant under high-stress fatigue, leading to a sharp deterioration in mechanical performance and a pronounced macroscopic decline in residual elongation.

**Figure 7:**
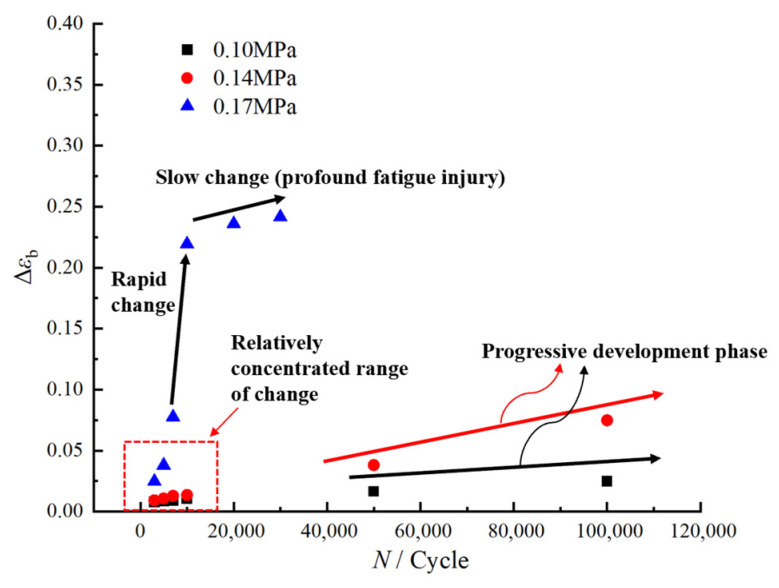
Variation in residual elongation decay ratio of HTPB propellant with loading conditions.

To further elucidate the evolutionary trend of residual elongation under fatigue loading, we analyzed the changes in the residual elongation decay ratio of the propellant under varying loading conditions. These data provide critical insights into the progression of material degradation, and the corresponding curve of residual elongation decay ratio versus loading conditions is presented in Figure 7, based on the measurements summarized in Table 4.

Overall, based on the analysis above, the evolution of residual elongation in HTPB propellant under different fatigue loading conditions can be categorized into three distinct regions: the “slow-change region”, the “gradual-decline region”, and the “rapid-decline region”. Within the “slow-change region”, residual elongation exhibits minimal fluctuation, indicating that HTPB propellant maintains robust mechanical performance under low-cycle fatigue conditions. In the “gradual-decline region”, residual elongation progressively decreases, suggesting that under medium-to-low stress fatigue, the continuous accumulation of cyclic loading leads to fatigue damage within the HTPB propellant, resulting in a gradual loss of plastic deformation capacity. In the “rapid-decline region”, residual elongation decreases sharply, indicating that high-stress fatigue causes rapid damage accumulation, severe deterioration of its mechanical properties, and accelerated material failure.

To quantitatively analyze the evolution of residual elongation in HTPB-based solid propellants under fatigue loading, we propose a three-region classification method based on the rate-of-change threshold approach. This method defines three distinct regions—slow-change, gradual-decline, and rapid-decline—by quantifying the correlation between residual elongation and the number of loading cycles. The regions are delineated using a threshold for the rate of change in residual elongation per cycle, reflecting the material’s damage evolution at different stages of fatigue. In this approach, the “instantaneous rate of change” of residual elongation is defined as the change in residual elongation per unit of loading cycle. It is calculated using Formula (3):

(3) $$k_{n}=\left|\frac{\varepsilon_{i,N}-\varepsilon_{i,0}}{N}\right|\times 100\%$$

where $\varepsilon_{i,N}$ is the residual elongation at the *N*-th loading cycle, $\varepsilon_{i,0}$ is the initial value of the elongation, and *N* is the number of loading cycles.

The fatigue process is classified as follows:

Slow-Change Region: In this region, the number of loading cycles is lower than 30,000, and the instantaneous change rate of residual elongation is below the threshold of 0.00015% (determined based on the magnitude of the instantaneous change rate of initial residual elongation). The residual elongation fluctuates minimally, indicating slow damage accumulation, and the propellant’s performance remains relatively stable, with fatigue damage remaining minimal.

Rapid-Decline Region: In this region, the number of loading cycles is still lower than 30,000, but the instantaneous rate of change in residual elongation significantly exceeds the threshold of 0.00015%. The residual elongation decreases sharply, reflecting rapid damage accumulation and significant deterioration of the material’s internal structure, which in turn leads to a severe decline in propellant performance.

Gradual-Decline Region: After 30,000 cycles, although the rate of change in residual elongation falls below the threshold, the residual elongation continues to decrease progressively due to the high number of cycles. In this region, the HTPB propellant experiences a gradual decline in performance as damage accumulates over extended cycling.

The accurate quantification of these regions can be compromised by variations in material properties and testing conditions, such as the filler content, temperature, or loading frequency. These factors may introduce deviations from the strict thresholds. In this study, the classification is primarily used to provide a systematic framework for understanding fatigue evolution rather than to define rigid, universally applicable boundaries. Its purpose is to capture key trends in residual elongation across the fatigue life of different HTPB-based propellant formulations.

### 3.2. Analysis of the Influence of Mesostructural Changes on Residual Elongation

**Figure 8:**
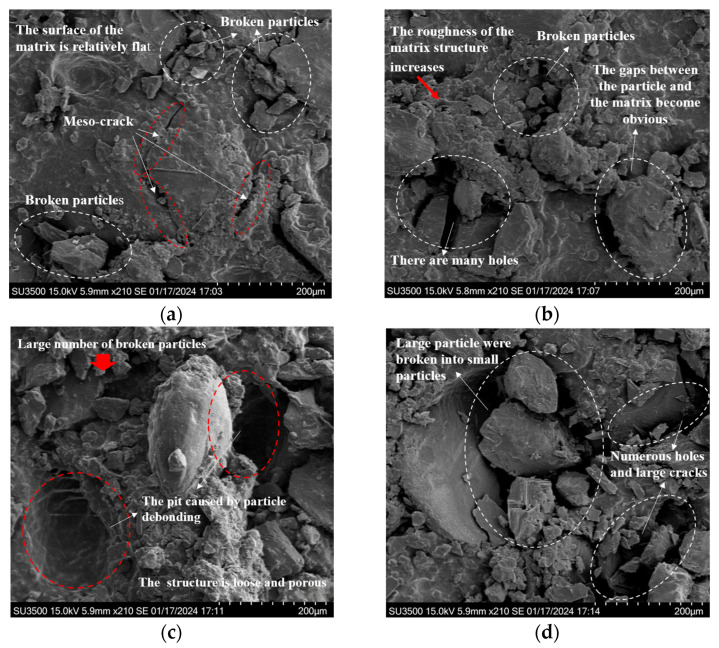
Mesostructural features of HTPB propellant specimens under the maximum loading stress of 0.14 MPa after varying numbers of loading cycles: (**a**) 5000; (**b**) 10,000; (**c**) 50,000; (**d**) 100,000.

**Figure 9:**
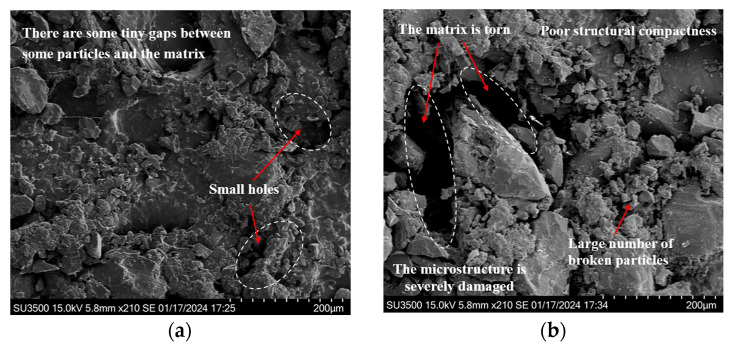
Mesostructural appearance after 10,000 cycles at different maximum loading stresses: (**a**) 0.1 MPa; (**b**) 0.17 MPa.

Building on the findings in Section 3.1, the evolution of the residual elongation of HTPB propellants under various fatigue load conditions can be clearly delineated. To further investigate the mechanisms through which changes in the mesostructure during the fatigue process affect residual elongation, and to clarify the intrinsic relationship between mesostructural damage behavior and the degradation of mechanical properties, this study characterizes the post-fatigue loading mesostructure of HTPB propellant samples. High-resolution observations of the mesostructure were conducted using scanning electron microscopy (SEM). Given the weak conductivity of the HTPB propellant, samples were sputter-coated with gold before imaging to prevent charge accumulation and image distortion. The resulting mesostructural images are presented in Figure 8 and Figure 9.

Figure 8 illustrates the surface mesostructural characteristics of HTPB propellant samples subjected to fatigue loading under a maximum loading stress of 0.14 MPa across various loading cycles. As observed, significant changes in the mesostructure are observed at different stages of fatigue, indicating that the material’s damage mechanisms evolve with the number of loading cycles.

Specifically, Figure 8a shows that, after 5000 loading cycles, a certain number of microcracks appear. These cracks are small, exhibit limited propagation, and have an irregular distribution. They have not yet formed a cohesive crack network. At this stage, the material’s mesostructure remains relatively intact, with only localized plastic deformation and fragmentation around the crack regions, and the surface remains mostly smooth. This indicates that the damage is still in the initial accumulation phase, with low severity, allowing the HTPB propellant to maintain high residual elongation under these conditions.

As the number of loading cycles increases to 10,000, as shown in Figure 8b, the number of voids in the propellant’s structure increases significantly. Two main mechanisms drive this phenomenon: firstly, prolonged fatigue loading induces phase transformations and dislocation movements within the material’s mesostructure, leading to atomic diffusion and reorganization, which cause voids to form; secondly, the dynamic expansion and intersection of microcracks cause stress concentrations at the crack tips, which ultimately result in the formation of closed voids. Additionally, some particle fragmentation becomes visible on the surface. The presence of gaps at the particle–matrix interface and the discontinuities in surface morphology indicate the development of a complex three-dimensional crack network. Compared to the state after 5000 cycles, the mesostructural integrity is significantly compromised after 10,000 cycles. These changes indicate that, with increasing loading cycles, the material’s load-carrying capacity declines, reducing its plastic deformation ability and leading to a decrease in residual elongation.

At 50,000 loading cycles, as shown in Figure 8c, larger pits appear on the surface of the HTPB propellant. This is due to internal stresses induced by cyclic loading that exceed the interfacial bonding strength, causing filler particles to detach from the matrix and leaving pit-like depressions. The crack width increases considerably, and numerous fragmented particles are scattered across the surface, further degrading the material’s mesostructural integrity. These observations demonstrate that, under high-cycle fatigue, the “dewetting” phenomenon in HTPB propellants becomes more pronounced, exacerbating the material’s structural damage. As the damage intensifies, the material’s capacity to maintain its structure decreases, leading to a marked reduction in residual elongation.

After 100,000 loading cycles, as seen in Figure 8d, fatigue damage has significantly advanced. Larger particles have fragmented into many smaller pieces, resulting in an extremely rough surface. Apparent gaps are visible between the particles and the matrix, accompanied by numerous pores and signs of tearing damage in the matrix. These features clearly indicate that fatigue loading has caused substantial structural damage to the HTPB propellant, leading to a considerable reduction in residual elongation.

Overall, under the same maximum loading stress, as the number of loading cycles increases, the mesostructure of the HTPB propellant undergoes continuous degradation. Cracks expand and connect, the number and size of voids increase, and particle–matrix interfaces experience increasing separation and damage. This ongoing disruption to material continuity reduces the HTPB propellant’s ability to undergo plastic deformation, causing a steady decline in residual elongation.

To further explore the impact of fatigue loads on residual elongation from a mesostructural perspective, this study also presents mesostructural images of HTPB propellant samples after 10,000 fatigue loading cycles under different maximum loading stresses of 0.1 MPa, 0.14 MPa, and 0.17 MPa (corresponding to Figure 8b and Figure 9a,b).

Analysis of Figure 9a shows that when the maximum loading stress is 0.1 MPa, the particle–matrix interface remains well-bonded, with no noticeable detachment or fragmentation of particles. The fatigue cracks are limited in number and length, with minimal propagation. The interior of the material shows only a few minute voids. This suggests that, under lower loading stresses, fatigue-induced damage is relatively minor, with a slow accumulation of internal damage. The material’s mesostructure remains relatively intact, and the HTPB propellant retains a high residual elongation, demonstrating good mechanical stability under these conditions.

When the maximum loading stress is increased to 0.14 MPa, as shown in Figure 8b, compared to the condition at 0.1 MPa, the bonding strength between the HTPB propellant particles and the matrix decreases. Gaps appear at the boundaries of some particles, indicating partial debonding and separation. Simultaneously, there is a noticeable increase in the number and size of voids within the material, along with localized fragmentation of particles. This leads to some disruption to the homogeneity of the mesostructure. Due to the interconnected expansion of cracks and the reduction in phase boundary bonding strength, the HTPB propellant becomes more susceptible to stress concentrations during tensile deformation, resulting in a decrease in residual elongation compared to the state at 0.1 MPa. However, as severe penetration damage has not yet occurred, the material still retains some load-bearing capacity, and therefore, the decline in residual elongation is not as substantial.

When the maximum loading stress reaches 0.17 MPa, as shown in Figure 9b, the mesostructure of the HTPB propellant exhibits pronounced degradation. Microscopic examination reveals a significant increase in the number of interconnected cracks, forming a dense crack network. This suggests that under high-stress conditions, the rate of crack propagation accelerates significantly, leading to the rapid accumulation of internal damage. Additionally, the mesostructure shows several signs of deterioration, including extensive particle fragmentation, disruption to the bonds between particles and the matrix, an increase in the size and interconnection of pores, and the tearing and destruction of the matrix. These combined processes cause the previously compact mesostructure to lose its integrity. Under such severe damage conditions, the material’s plastic deformation capacity drastically decreases, resulting in a substantial reduction in residual elongation and a significant decline in mechanical properties.

In summary, during the fatigue loading process in HTPB propellants, there is a significant positive correlation between stress levels and both the extent of material damage and the degradation of properties. Higher maximum loading stresses intensify the complexity and concentration of internal stress fields, thereby facilitating the emergence of numerous microcracks, holes, and particle fragmentation. These mesostructural damages continuously weaken the HTPB propellant’s capacity for plastic deformation, consequently leading to a progressive decrease in residual elongation and a progressive degradation of mechanical performance.

## 4. Model and Validation

### 4.1. Model Construction

In practical applications, accurately characterizing the evolution of residual elongation under fatigue loads in HTPB propellants is essential in predicting the HTPB propellant’s performance and longevity. To achieve this, this study adopts a concise and unified expression to describe the variation in the decay ratio of residual elongation under different loading conditions during the fatigue process. Inspired by the classic Williams–Landel–Ferry (WLF) theory [29], which constructs a master curve through shifting response data, this study extends the concept to the fatigue analysis of solid propellants using stress–cycle superposition.

WLF theory, which is originally based on the time–temperature superposition principle, describes the viscoelastic behavior of materials by shifting response data along the time axis. In this study, a similar approach is applied to fatigue analysis, where higher stress levels correspond with fewer cycles being required to reach a given damage state. In comparison, lower stress levels correspond with more cycles being required. This establishes an equivalence between stress and the number of loading cycles, which enables the superposition of fatigue data under different stress levels.

Through the mathematical fitting of this master curve, an evolutionary model for the residual elongation decay ratio is developed as a function of the maximum loading stress and the number of cycles. This model serves as a quantitative tool for predicting fatigue life and assessing the effects of varying loading conditions on material behavior.

The Time–Stress Superposition Principle (TSSP) [30,31,32] is a well-established concept in polymer mechanics, particularly in materials such as solid propellants and rubber-like substances. Under a constant-frequency alternating cyclic loading, the number of cycles *N* is equal to the product of loading time *t* and frequency *f*. This means that, with a fixed loading frequency *f*, the number of loading cycles *N* can be considered equivalent to the time *t*. This leads to the stress–cycle superposition principle, which suggests that the mechanical response at higher stress for fewer cycles can be equivalently mapped to a lower stress for more cycles, using an experimentally determined stress shift factor.

This principle allows for the shifting of mechanical response curves, such as curves of residual elongation, along the loading cycle axis, thereby constructing a master curve. The master curve represents the material’s response over a wide range of conditions, from short-term high-stress behavior to long-term low-stress behavior, thereby reducing the need for extensive experimental testing. In the context of solid propellants, this principle facilitates the equivalence between stress levels and loading cycle numbers, enabling the construction of a master curve that illustrates the decay of residual elongation across various loading conditions. By superimposing fatigue data from different stress levels, this study develops the master curve to represent the decay ratio of residual elongation. This technique provides a unified framework for quantifying fatigue damage and predicting the HTPB propellant’s behavior across different stress levels, ultimately enabling more efficient fatigue life predictions.

The analogy between temperature–frequency and stress–cycle mechanical behavior suggests that both are controlled by unified underlying time and load accumulation scales. Temperature affects molecular motion, controlling the “effective time scale” for dynamic loading, while stress influences microdamage accumulation, controlling the “effective time scale” for load accumulation.

This “scale equivalence” allows WLF-like shift factors to superpose fatigue data, creating a master curve for fatigue analysis in HTPB propellants. This analogy assumes periodic load variations and an ideal, homogeneous material, excluding non-stationary responses and defects.

**Figure 10:**
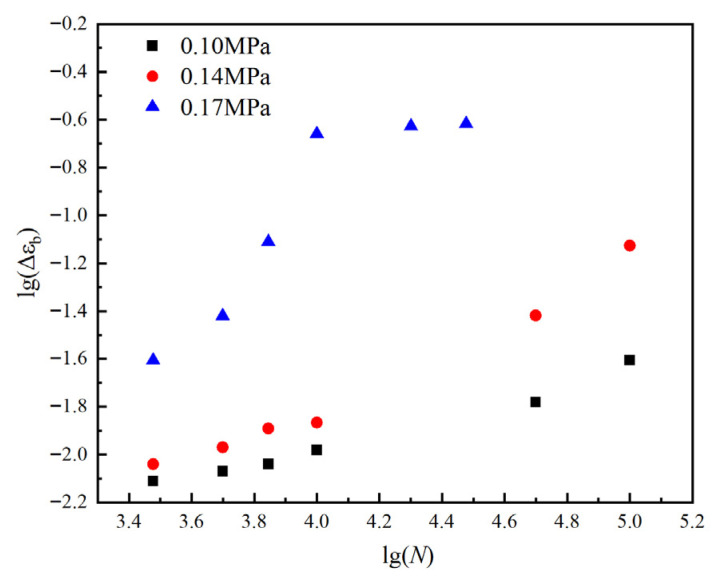
Variation in residual elongation decay ratio of HTPB propellant under different loading conditions (in logarithmic coordinates).

To develop the master curve, this study presents the data more clearly and intuitively by logarithmically transforming both the horizontal axis (number of loading cycles) and the vertical axis (decay ratio of residual elongation), as shown in Figure 7. The transformed curves, illustrating the variation in the residual elongation decay ratio under different loading conditions, are presented in Figure 10.

As is evident from Figure 10, the overall trend of the residual elongation decay ratio across various loading conditions appears as a nearly linear increase, which supports the consistency necessary for the horizontal shifting of the data. By appropriately adjusting the horizontal shift (number of loading cycles), it is possible to superimpose curves from different stress levels, aiming to construct a comprehensive master curve that describes the evolution of the residual elongation decay ratio in HTPB propellants.

In the translation process of data curves, the selection of appropriate loading benchmark conditions is critical. To ensure the master curve accurately represents the physical characteristics of the actual test and mirrors the evolution of the HTPB propellant’s mechanical properties, it is imperative to scrutinize the effects of translation across various benchmark conditions. The data presented in Table 2 indicate that at a maximum loading stress of 0.14 MPa, the HTPB propellant specimen reaches its fatigue limit at approximately 1.5 × 10^5^ cycles. Utilizing this stress condition as a benchmark for translation results in a calculated maximum number of equivalent loading cycles that exceeds the actual fatigue limit loading cycles, which is contradictory to the experimental results. In addition, employing the data obtained at 0.17 MPa as the benchmark results in considerable fluctuations in the residual elongation decay ratio within the lower range of equivalent loading cycles, which also conflict with the empirical results. A comprehensive analysis suggests that selecting the data curve obtained under a maximum loading stress of 0.1 MPa as the benchmark for constructing the master curve of equivalent loading cycles represents the most rational approach in this study.

**Figure 11:**
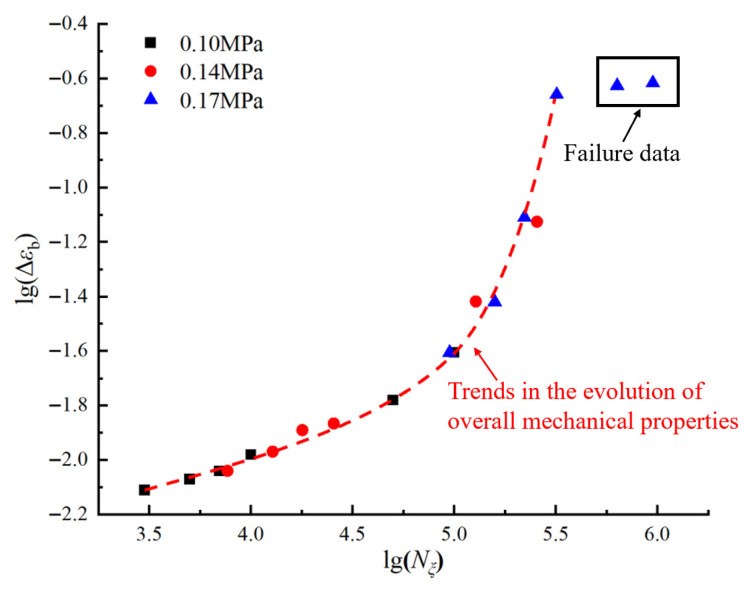
The evolution trend of the decay ratio of propellant residual elongation with equivalent loading cycles (in logarithmic coordinates).

Figure 11 employs a logarithmic coordinate system to show the evolution trend of the residual elongation decay ratio under 0.1 MPa benchmark stress with the equivalent number of loading cycles. This trend is obtained by horizontally translating the data points of residual elongation decay ratio at different numbers of loading cycles for the maximum loading stresses of 0.14 MPa and 0.17 MPa. The conversion relationship between the equivalent number of loading cycles and the actual number of loading cycles is given in Equation (4):

(4) $$lg\left(N_{\xi }\right)=lg\left(N\right)+lg\left(\alpha_{\varepsilon }\right)$$

Here, *N* represents the actual number of loading cycles, $N_{\xi }$ represents the equivalent number of loading cycles, and $lg\left(\alpha_{\varepsilon }\right)$ signifies the horizontal logarithmic translation amount of data points. The factor $\alpha_{\varepsilon }$ is termed the equivalent factor for the decay ratio curve of the residual elongation. The equivalent factor $\alpha_{\varepsilon }$ serves to normalize fatigue damage under different maximum loading stresses, enabling the decay of residual elongation to be unified onto a single master curve at a reference stress. Its physical basis lies in two aspects: higher stress accelerates microscopic damage, leading to greater residual elongation decay per cycle, and different stress levels require different cycle numbers to reach the same damage state. The factor $\alpha_{\varepsilon }$ mathematically accounts for these effects, converting high-stress, low-cycle damage into the corresponding equivalent number of cycles at the reference stress, thus providing a consistent framework to describe the intrinsic fatigue damage evolution in the material.

From the transformed curve, it can be observed that, at the stage when the decay ratio of the residual elongation corresponds to the black-framed area in Figure 11, combined with the above mesoanalysis, the mesostructure of the HTPB propellant has suffered severe damage, causing the material to enter a fatigue failure state and thus rendering it unsuitable for practical applications. Therefore, such failure data should be excluded when constructing the master curve. Given the inherent scatter in the tests, data with significant deviation should also be removed.

**Figure 12:**
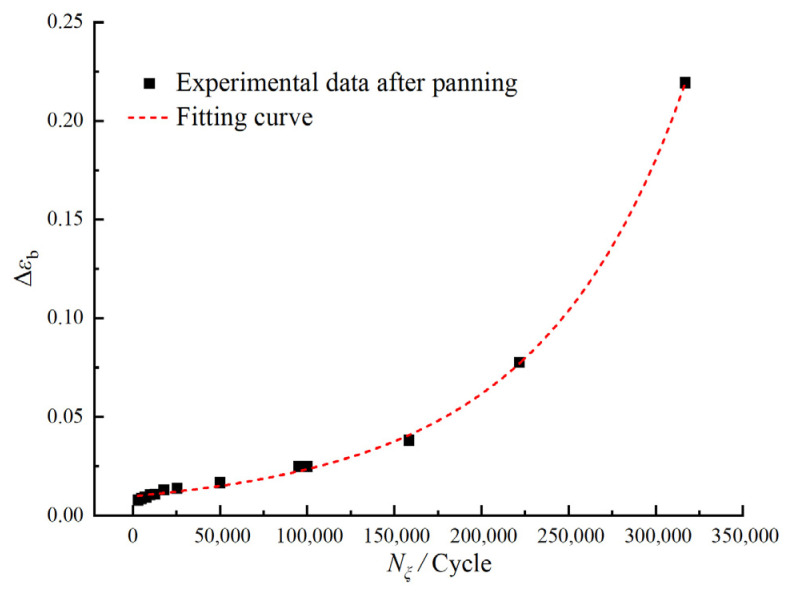
Master curve of decay ratio of propellant residual elongation with equivalent loading cycles.

After removing failure data and completing the data screening process, the “master curve” of the HTPB propellant residual elongation decay ratio with respect to the equivalent number of loading cycles is shown in Figure 12 (linear coordinate system).

By utilizing Origin Pro 2021 software to perform non-linear fitting on the master curve in Figure 12, we obtained the fitting equation shown in Equation (5), with a coefficient of determination *R*^2^ of 0.999.

(5) $$\Delta \varepsilon_{b}={\left(1.62209-1.42076\times 10^{-6}\cdot N_{\xi }\right)}^{-9.57304}$$

During the construction of the master curve, the amount of shift required for data points corresponding to different maximum loading stresses, quantified as $lg\left(\alpha_{\varepsilon }\right)$, varies, indicating that the equivalent factor $\alpha_{\varepsilon }$ is related to the maximum loading stress. Analysis using Origin software reveals that, for a maximum loading stress of 0.14 MPa, the mean value of $lg\left(\alpha_{\varepsilon }\right)$ is approximately 0.408, while for a maximum loading stress of 0.17 MPa, it averages about 1.501. Non-linear fitting of this data yields the relationship expression between the equivalent factor $\alpha_{\varepsilon }$ and the maximum loading stress $\sigma_{max}$, as shown in Equation (6), with *R*^2^ = 0.999.

(6) $$lg\left(\alpha_{\varepsilon }\right)=-0.12765+0.00336\cdot e^{36.37418\cdot \sigma_{max}}$$

Further integrating Equations (4)–(6) yields the comprehensive mathematical expression for the evolution model of the residual elongation, detailing the decay ratio of the residual elongation as a function of different stress levels and numbers of loading cycles.

(7) $$\Delta \varepsilon_{b}={\left(1.62209-1.05894\times 10^{-6+0.00336\cdot e^{36.37418\cdot \sigma_{max}}}\cdot N\right)}^{-9.57304}$$

In this equation, $\Delta \varepsilon_{b}$ represents the decay ratio of the residual elongation, $\sigma_{max}$ denotes the maximum loading stress, and *N* signifies the number of loading cycles.

### 4.2. Model Validation and Sensitivity Analysis

This study extends the application of the Williams–Landel–Ferry (WLF) theory to the fatigue analysis of solid propellants by integrating the stress–cycle superposition approach. The method allows for the construction of a unified master curve that quantifies the decay ratio of residual elongation under various loading conditions, providing a comprehensive framework for both fatigue life prediction and material performance assessment.

**Figure 13:**
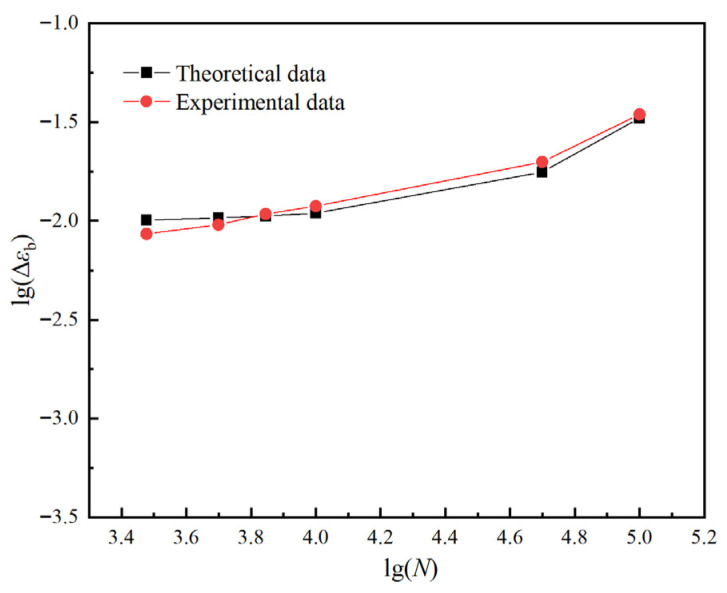
Comparison of theoretical and experimental results.

To validate the accuracy of the proposed evolutionary model, fatigue tests were conducted under a maximum loading stress of 0.12 MPa over various loading cycles, as outlined in Figure 13, which compares the model’s theoretical predictions with the corresponding experimental results. To provide a more precise and more intuitive view of the variations and discrepancies between the two datasets during the fatigue process, a logarithmic transformation was applied to the data. As shown in Figure 13, there are deviations between the predicted and experimental results throughout the loading process. These discrepancies can be attributed to several factors. Firstly, the initiation and propagation of microcracks in the HTPB propellant, along with the repeated opening and closing of the “dewetting” interface, are highly complex processes [33] that the model cannot fully capture. Secondly, uncertainties related to experimental apparatus precision, specimen preparation, and environmental conditions also contribute to the differences observed. Nevertheless, the overall agreement between experimental data and model predictions demonstrates that the evolutionary model effectively describes the decay of residual elongation in HTPB propellants under cyclic loading at 323 K.

To further assess the model, a sensitivity analysis was conducted to evaluate the influence of maximum loading stress and the number of loading cycles on residual elongation decay. A single-parameter perturbation method was used, in which one parameter was varied while the other was held constant. Specifically, when examining the effect of maximum stress, the cycle number was fixed at 10,000, and calculations were performed for different stress levels. Conversely, when analyzing the impact of cycle number, the maximum stress was fixed at 0.12 MPa, and other loading cycle numbers were considered.

The findings from the sensitivity analysis were essential in developing a unified master curve for residual elongation decay. By combining the effects of maximum stress and cycle number, the master curve was constructed to reflect the whole degradation process of HTPB propellants under cyclic loading. This curve serves as an important tool for predicting fatigue life and assessing HTPB propellant’s performance. It enables a better understanding of how stress and cycle number interact to influence material degradation, aiding in the optimization of loading conditions to extend the service life of HTPB propellants.

To quantify the sensitivity of residual elongation decay to maximum stress and cycle number, the sensitivity coefficient was calculated using the relative rate of change as follows:

(8) $$S=\frac{\Delta \Delta \varepsilon_{b}/\bar{\Delta \varepsilon_{b}}}{\Delta p/\bar{p}}$$

where $\Delta \Delta \varepsilon_{b}$ is the change in residual elongation decay, $\bar{\Delta \varepsilon_{b}}$ is the average value of decay, Δp is the change in the model parameter (either $\sigma_{max}$ or N), and $\bar{p}$ is the average value of that parameter.

For the maximum applied stress, increasing $\sigma_{max}$ from 0.1 MPa to 0.12 MPa resulted in a sensitivity coefficient of approximately *S* ≈ 0.733, and, from 0.12 MPa to 0.16 MPa, the sensitivity coefficient increased sharply to *S* ≈ 4.235. This demonstrates a strong non-linear sensitivity to applied stress. In comparison, regarding the cycle number, increasing *N* from 5000 to 10,000 yielded *S* ≈ 0.319, and, from 10,000 to 50,000, *S* increased to 0.868. These results indicate that the effect of cycle number on elongation decay is more gradual and less influential than the impact of the maximum loading stress.

In summary, the sensitivity analysis highlighted that the maximum applied stress is the primary driver of residual elongation decay, while the cycle number, although still relevant, plays a secondary role. These insights were crucial in the development of the master curve for predicting the fatigue life of HTPB propellants under cyclic loading conditions. This unified approach provides a comprehensive framework for understanding the HTPB propellant’s degradation and optimizing loading conditions to enhance the longevity of HTPB propellants in various aerospace applications.

## 5. Discussion

Research on solid propellant materials under fatigue loads has evolved from early models focusing on stress–strain responses under uniaxial fatigue to more complex investigations into multiaxial and thermo-mechanical fatigue, with an emphasis on macroscopic performance parameters such as modulus degradation. Recent studies have shifted toward elucidating the underlying fatigue damage mechanisms, using advanced in situ techniques and multi-scale simulations, to explore how microstructural changes, such as interface debonding, affect macroscopic properties. Despite progress, challenges remain in extreme service conditions, and further studies on residual elongation, particularly in HTPB-based composites, are needed to enhance our understanding of fatigue behavior.

### 5.1. Implications for Rocket Motor Design and Durability

The fatigue loading process of HTPB propellants reveals a significant positive correlation between stress levels and the extent of material damage, as well as the degradation of mechanical properties. As the maximum loading stresses increase, the internal stress fields within the material become more complex and concentrated, promoting the development of microcracks, voids, and particle fragmentation. These mesostructural changes progressively impair the material’s macroscopic capacity for plastic deformation, resulting in a continuous decrease in residual elongation and an overall decline in mechanical performance. This damage accumulation follows a non-linear trajectory, wherein the rate of mechanical property degradation accelerates markedly once a critical stress threshold is exceeded. This behavior highlights the intricate relationship between stress, material damage, and performance degradation under fatigue loading.

Our findings have clear implications regarding the long-term performance and safety of solid rocket motors. By integrating the observed degradation mechanisms into predictive models, engineers can more accurately assess how HTPB propellants, and, by extension, rocket motors, will perform under various fatigue and stress conditions over time. These models, which can incorporate factors such as stress–cycle history and damage accumulation laws, will enable more precise predictions regarding the life expectancy of rocket motors, allowing for better maintenance scheduling and timely intervention. This predictive capability is crucial in ensuring the safety and reliability of rocket motors in operational environments, where fatigue accumulation is inevitable and performance deterioration could pose significant risks. Moreover, establishing quantitative relationships between measurable material properties (e.g., modulus reduction, crack density) and remaining service life can form the basis for condition-based monitoring strategies.

Furthermore, our study highlights the need to consider thermomechanical cycling effects in the performance analysis of solid propellants. In real-world applications, temperature fluctuations can exacerbate the fatigue degradation process, accelerating the loss of mechanical properties and reducing the overall service life of the propellant. The differential thermal expansion between the solid filler particles and the polymeric binder matrix induces additional internal stresses during temperature cycles, which synergize with mechanical fatigue loads. Incorporating temperature variations into long-term performance simulations will provide a more comprehensive understanding of how thermal and mechanical fatigue interact to influence material degradation. Therefore, future experimental work should focus on characterizing the propellant’s behavior under combined thermomechanical cycling conditions to calibrate and validate such advanced models. Investigating the combined effects of these factors is essential in the design of more resilient and reliable propulsion systems. By addressing the synergistic impact of thermal and mechanical loading, future research can offer valuable insights that will help engineers optimize rocket motor design and improve their operational performance over extended periods. This holistic approach is paramount for advancing the safety and longevity of solid rocket motors in demanding aerospace applications.

### 5.2. Relationship Between Laboratory-Scale and Full-Scale Propellant Behavior

Although the results of this study provide valuable insights into the fatigue behavior of HTPB propellants on the laboratory scale, translating these findings into full-scale rocket motor applications means addressing several scale-dependent effects that can materially change damage evolution and lifetime predictions. The smaller specimen size used in our experiments results in simpler, more uniform stress fields and rapid thermal equilibration, which are not representative of a full-scale grain, where complex geometry, boundary constraints, gravity, ignition shocks, and operational loads induce highly non-uniform stress distributions and local stress concentrators (e.g., hole corners, bond lines, and shell interfaces). Moreover, the large radial dimensions of full grains create significant temperature gradients during operation; spatially varying temperatures induce differential thermal strains and additional thermal stresses that accelerate local damage accumulation. Material heterogeneity is also amplified at scale: casting, curing, and handling of large charges can introduce local density variations, voids, and process-related defects that reduce local strength and alter fatigue crack initiation statistics relative to laboratory specimens.

To improve transferability, we propose three complementary corrections. Firstly, finite-element analysis (FEA) of full-scale geometries (including gravity, ignition shock, and shell constraints) should be employed to calculate stress concentration factors, and a stress-correction term should be incorporated into the evolution model so that local amplified stresses, rather than nominal stresses, drive damage evolution. Secondly, thermal gradients should be addressed by coupling transient heat-transfer simulations of engine thermal environments with temperature-dependent material behavior measured across the laboratory temperature range; the resulting temperature correction term will capture additional thermally induced stresses that alter damage. Thirdly, material heterogeneity should be addressed through extensive sampling and mechanical characterization across large grains and batches to quantify property variability and defect statistics. These data will inform a process correction factor to compensate for differences in mixing, casting, and curing between laboratory specimens and production-scale grains.

Finally, we will iteratively calibrate and validate the corrected model using subscale tests that reproduce identified stress concentrators and thermal gradients, followed by targeted full-scale or representative section tests where feasible. Together, stress, temperature, and process correction factors—derived from FEA, thermal simulations, and large-scale sampling—will increase our confidence in the evolution model reliably predicting damage and residual elongation in operational rocket motors.

### 5.3. Multi-Scale Fatigue Analysis of Solid Propellants

This study highlights the significance of a multi-scale approach to understanding fatigue in HTPB propellants. Our mesostructural observations reveal that microstructural changes, such as microcracks and particle fragmentation, evolve under cyclic loading and exhibit a strong correlation with macroscopic residual elongation decay. This suggests that mesostructural degradation plays a crucial role in fatigue response.

The three-region classification of residual elongation (slow-change, gradual-decline, rapid-decline) offers a valuable framework. However, its boundaries are influenced by factors such as filler content, temperature, and loading frequency. For instance, higher filler content shortens the slow-change region, while elevated temperatures shift the gradual-decline region to lower cycles.

Due to equipment limitations and sample constraints, we could not perform precise microscale quantification (e.g., crack density, porosity). Current micro-CT-based methods are hindered by accuracy and cost, and external institutions were unable to test solid propellants due to safety concerns. Despite this, our qualitative SEM observations provide valuable insights, correlating with macroscopic trends in residual elongation.

To establish a clear quantitative link between microstructural features and residual elongation, and to optimize HTPB propellant formulations and processes, future work will focus on enhancing experimental capabilities. We plan to acquire high-resolution scanning electron microscopes (SEM) and micro-computed tomography (micro-CT) systems. Additionally, we will collaborate with institutions equipped with advanced characterization techniques for energetic materials. This will allow us to incorporate quantitative analyses, such as crack density, void fraction, and particle size distributions, to strengthen our understanding of the relationship between microstructural observations and macroscopic material behavior.

### 5.4. Theoretical Parallelism Between WLF Theory and Stress–Cycle Relationships

A key element of this work is the analogy between the time–temperature superposition principle (TTSP) in WLF theory and the stress–cycle relationship in fatigue analysis. In WLF theory, temperature regulates molecular motion, with higher temperatures leading to increased mobility and faster relaxation processes. This results in equivalence between short-term mechanical responses at high temperatures (high-frequency loading) and long-term responses at low temperatures (low-frequency loading), enabling the construction of a master curve across different temperature ranges.

Similarly, stress controls the rate of damage accumulation in fatigue analysis. As stress increases, the material undergoes more rapid degradation, leading to shorter fatigue life, analogous to the faster response seen at higher temperatures. The stress shift factor functions similarly to the temperature shift factor in WLF theory, enabling the construction of a fatigue master curve across varying stress levels.

Both frameworks share a common principle: adjusting a single variable (temperature or stress) to control molecular motion and achieve equivalence in mechanical responses across different time or cycle scales. This analogy provides a unified approach to elucidating material behavior under various loading conditions and strengthens the theoretical foundation of our study.

## 6. Conclusions

This study explores the evolution of the mechanical properties of HTPB propellants under fatigue loading and the underlying mechanisms. Based on the experimental results, the evolution of the propellant’s residual elongation and the characteristic changes in its microstructural scale were analyzed in detail, and a corresponding evolution model was developed to describe these changes. The validity of this model was confirmed through experimental validation, providing new insights into material degradation behavior. The novelty of this study lies in the use of an integrated approach that links macroscopic mechanical performance with microscopic structural damage, thereby offering a more comprehensive understanding of HTPB propellant behavior under fatigue conditions. Additionally, a predictive framework for its long-term performance under different loading conditions was established. The study draws the following conclusions:

(1) This study proposes a three-region classification method to quantitatively analyze the evolution of residual elongation in HTPB propellants under fatigue loading. By leveraging a rate-of-change threshold approach, the method defines three distinct regions—slow-change, gradual-decline, and rapid-decline—based on the correlation between residual elongation and the number of loading cycles. These regions are delineated by thresholds for the instantaneous rate of change in residual elongation, reflecting the material’s damage evolution at different fatigue stages. The slow-change region, characterized by minimal damage accumulation, indicates relatively stable performance, while the rapid-decline region signifies a significant deterioration in the propellant’s internal structure, leading to a sharp decline in performance. The gradual-decline region reflects the continued, though slower, degradation of the propellant after extensive cycling.
  This classification provides a systematic framework for understanding fatigue evolution, offering a clearer insight into the material’s behavior under cyclic loading. It also serves as a valuable tool to track the degradation process of different propellant formulations, allowing for the identification of critical stages where performance may significantly decline. While the exact thresholds for classification may vary due to factors such as the material composition, temperature, or loading frequency, the proposed method is effective in capturing the key trends across the fatigue life of solid propellants. Overall, this approach offers new perspectives on long-term performance prediction for solid propellants under fatigue conditions, guiding the optimization of their material properties and performance stability.
(2) At the mesoscopic level, higher maximum loading stresses and increased loading cycles lead to the expansion and interconnection of microcracks within the HTPB propellant. At the same time, there is an increase in the number and size of voids, separation at phase boundaries, and the degree of particle fragmentation. These phenomena progressively degrade the propellant’s continuity and integrity, causing a gradual reduction in its plastic deformation capacity and a continuous decline in residual elongation.
(3) The master curve depicting the decay ratio of the residual elongation was constructed, and, based on this curve, a residual elongation evolution model was established. The mathematical expression of the model is as follows:

(9) $$\Delta \varepsilon_{b}={\left(1.62209-1.05894\times 10^{-6+0.00336\cdot e^{36.37418\cdot \sigma_{max}}}\cdot N\right)}^{-9.57304}$$

The validation experiments showed that this model effectively captures the evolution of residual elongation in HTPB propellants under various maximum loading stresses and loading cycles at a testing temperature of 323 K. This validates the model’s ability to accurately describe the material’s mechanical behavior, providing a solid theoretical foundation for further studies on the macroscopic mechanical properties of this propellant.

## Figures and Tables

**Table 1:** Composition/component information of HTPB propellant.

Component	Role	Mass/%	Density/g·cm^−3^	CAS No.
HTPB	Adhesive	12%	0.95	69102-90-5
AP	Oxidant	69%	1.95	7790-98-9
Al	Fuel	18%	2.70	7429-90-5
Other	-	1%	-	-

**Table 2:** Experimental results for propellant fatigue life at various stress levels.

***σ_max_*** **/MPa**	0.10	0.14	0.17
***N_f_*** **/cycle**	1.27 × 10^6^	1.53 × 10^5^	4.76 × 10^4^

**Table 3:** Fatigue testing protocol for HTPB propellant.

*σ_max_*/MPa	*N*					
0.10	3000	5000	7000	10,000	50,000	100,000
0.14	3000	5000	7000	10,000	50,000	100,000
0.17	3000	5000	70,000	10,000	20,000	30,000

**Table 4:** Residual elongation $\varepsilon_{i}$ and decay ratio of residual elongation $\Delta \varepsilon_{b}$ of HTPB propellant specimens under different fatigue conditions.

*σ*_max_	*N*	*ε_i_*	Δ*ε_b_*	*σ*_max_	*N*	*ε_i_*	Δ*ε_b_*	*σ*_max_	*N*	*ε_i_*	Δ*ε_b_*
0.10 MPa	3000	39.689%	0.778%	0.14 MPa	3000	39.633%	0.918%	0.17 MPa	3000	39.008%	2.480%
	5000	39.659%	0.851%		5000	39.571%	1.073%		5000	38.479%	3.802%
	7000	39.635%	0.913%		7000	39.485%	1.288%		7000	36.897%	7.758%
	10,000	39.581%	1.047%		10,000	39.455%	1.362%		10,000	31.229%	21.928%
	50,000	39.337%	1.658%		50,000	38.474%	3.815%		20,000	30.562%	23.595%
	100,000	39.007%	2.483%		100,000	37.011%	7.473%		30,000	30.341%	24.150%

## Data Availability

Data are contained within the article.
